# BacSeq: A User-Friendly Automated Pipeline for Whole-Genome Sequence Analysis of Bacterial Genomes

**DOI:** 10.3390/microorganisms11071769

**Published:** 2023-07-06

**Authors:** Arnon Chukamnerd, Kongpop Jeenkeawpiam, Sarunyou Chusri, Rattanaruji Pomwised, Kamonnut Singkhamanan, Komwit Surachat

**Affiliations:** 1 Division of Infectious Diseases, Department of Internal Medicine, Faculty of Medicine, Prince of Songkla University, Songkhla 90110, Thailand; arnonchukamnerd@hotmail.com (A.C.); sarunyouchusri@hotmail.com (S.C.); 2 Department of Biomedical Sciences and Biomedical Engineering, Faculty of Medicine, Prince of Songkla University, Songkhla 90110, Thailand; kongpop.je@gmail.com; 3 Division of Biological Science, Faculty of Science, Prince of Songkla University, Songkhla 90110, Thailand; rattanaruji.p@psu.ac.th; 4 Translational Medicine Research Center, Faculty of Medicine, Prince of Songkla University, Songkhla 90110, Thailand; 5 Division of Computational Science, Faculty of Science, Prince of Songkla University, Songkhla 90110, Thailand

**Keywords:** whole-genome sequencing, BacSeq, assembly, annotation, bioinformatics

## Abstract

Whole-genome sequencing (WGS) of bacterial pathogens is widely conducted in microbiological, medical, and clinical research to explore genetic insights that could impact clinical treatment and molecular epidemiology. However, analyzing WGS data of bacteria can pose challenges for microbiologists, clinicians, and researchers, as it requires the application of several bioinformatics pipelines to extract genetic information from raw data. In this paper, we present BacSeq, an automated bioinformatic pipeline for the analysis of next-generation sequencing data of bacterial genomes. BacSeq enables the assembly, annotation, and identification of crucial genes responsible for multidrug resistance, virulence factors, and plasmids. Additionally, the pipeline integrates comparative analysis among isolates, offering phylogenetic tree analysis and identification of single-nucleotide polymorphisms (SNPs). To facilitate easy analysis in a single step and support the processing of multiple isolates, BacSeq provides a graphical user interface (GUI) based on the JAVA platform. It is designed to cater to users without extensive bioinformatics skills.

## 1. Introduction

High-throughput sequencing (HTS) technologies have revolutionized the field of genomics by allowing researchers to analyze large quantities of genetic material in a relatively short amount of time [1,2]. Short-read sequencing (SRS) and long-read sequencing (LRS) are powerful tools to study the entire genomes of bacteria [2]. The sequence reads from these technologies are generated as a fastq file, which needs bioinformatics tools for further analysis. Command-line, web-based, and program-based tools are currently used for sequence analyses [3]. Among them, command-line tools provide maximum flexibility and are highly customizable, but require a higher level of technical expertise and may be more time-consuming for certain tasks. Web-based tools, on the other hand, are generally more user-friendly and accessible to users without extensive bioinformatics training, but may have limitations in terms of customization and flexibility. Program-based tools provide a balance between the two, offering a graphical user interface that is more accessible than command-line tools while still providing a high degree of flexibility.

In a previous study, Quijada et al., (2019) developed automated pipelines called TORMES for analyzing whole-genome sequencing (WGS) data of bacteria generated by Illumina platforms [4]. TORMES automates the bioinformatic analysis steps, including sequence quality filtering, de novo assembly, draft genome ordering against a reference, genome annotation, multi-locus sequence typing (MLST), searching for antibiotic resistance and virulence genes, and pan-genome comparisons. The pipeline can be used for any set of bacteria from any species and origin, and more extensive analyses for *Escherichia* and *Salmonella* can be enabled using the −g/− genera option. Once the analysis is finished, TORMES generates an interactive web-like report that can be opened in any web browser, and shared and revised by researchers in a simple manner. However, it should be noted that TORMES may not be suitable for all types of WGS data, and researchers probably consider using other inputs, such as short-read sequences and long-read sequences from different platforms, to obtain a more comprehensive understanding of their bacterial genomes. Additionally, many researchers may not have the necessary bioinformaticians to fully utilize TORMES or other sequencing analysis tools. We then aimed to generate and improve an easy-to-use automated pipeline for WGS and bioinformatics analyses of bacterial genomes, which is beneficial for non-bioinformatician users.

## 2. Materials and Methods

### 2.1. Bioinformatics Pipeline

BacSeq integrates several frequently used open-source bioinformatics tools to perform a single-step analysis including assembly, assembly quality evaluation, gene prediction, functional annotation, specific gene identification, and pan-genome analysis. The pipeline begins with loading compressed raw read files (.fastq.gz; accessed on 5 May 2023) and checking the quality via FastQC (http://www.bioinformatics.babraham.ac.uk/projects/fastqc/; accessed on 5 May 2023), and the results are imported into MultiQC [5] to generate summary reports. The trimming step is then performed using fastp [6] to remove the adapter sequence, cut low-quality bases, and trim all reads at the 5’ and 3’ ends. Next, SPAdes [7] is used for assembling the filtered sequences into contigs and scaffolds using various k-mer lengths.

Next, genome assembly assessment and completeness evaluation are performed using QUAST [8] and BUSCO [9], respectively. For the annotation process, Prokka [10] is called to identify genomic features of interest in the assembled genome. Functional annotation is then performed with eggNOG-mapper [11] which combines HMMER [12], DIAMOND [13], MMSEQS2 [14], and PRODIGAL [15] to search against several databases including Clusters of Orthologous Groups of proteins (COGs) [16], Gene Ontology (GO) [17], Protein family (PFAM) [18], and Kyoto Encyclopedia of Genes and Genomes (KEGG) [19]. Next, the downstream analysis to identify pathogenic-related genes starts by running the ABRicate pipeline (https://github.com/tseemann/abricate/; accessed on 5 May 2023) to search against several databases including the Comprehensive Antibiotic Resistance Database (CARD) [20], ResFinder [21], Antibiotic Resistance Gene-ANNOTation (ARG-ANNOT) [22], MEGARes [23], Virulence Factor Database (VFDB) [24], PlasmidFinder [25], and ISfinder [26]. Carbohydrate-active enzymes (CAZyme) and Clustered Regularly Interspaced Short Palindromic Repeats and CRISPR-associated proteins (CRISPR-Cas) are then searched via automated CAZyme annotation [27,28] and CRISPRCasFinder [29], respectively.

**Figure 1:**
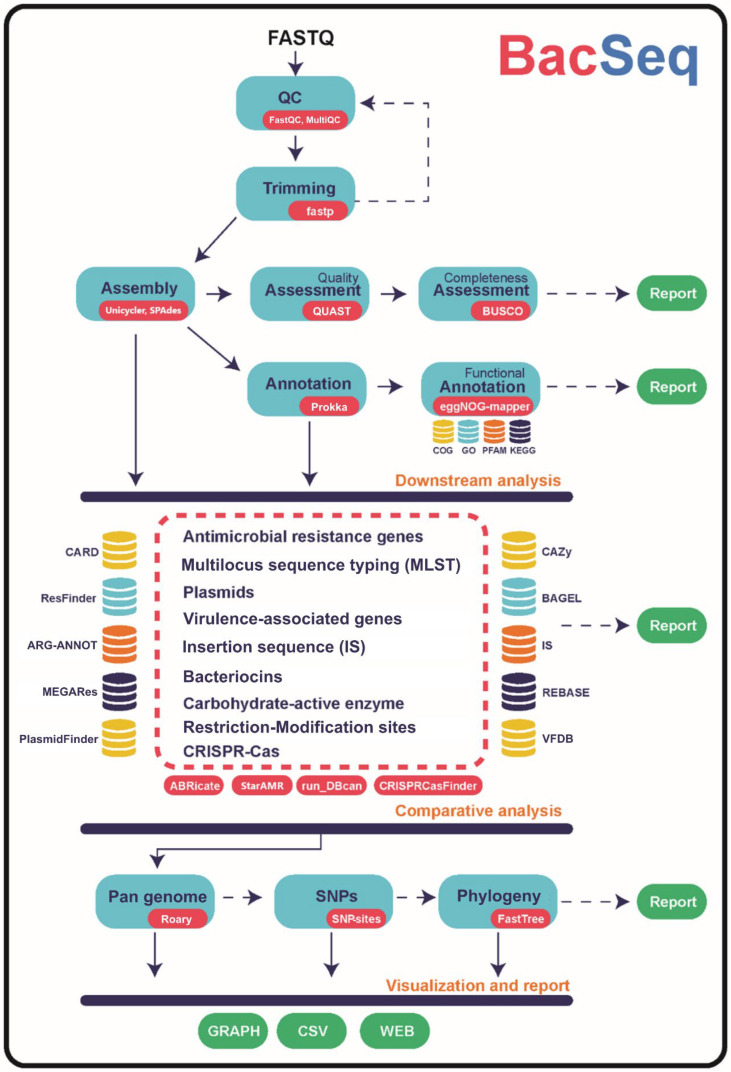
Bioinformatics workflow of BacSeq analysis steps.

A pan-genome analysis was then performed by Roary [30] to identify the core and accessory genes from a collection of assembled genomes. Single-nucleotide polymorphisms (SNPs) of core genes are called by SNP-sites [31] and constructed the phylogenetic tree using FastTree [32]. All analysis reports are finally generated by combining all results into web format and Comma-Separated Values (CSV) files. The overall bioinformatics workflow is presented in Figure 1.

### 2.2. Requirements

BacSeq is a JAVA-based application for analyzing WGS data using paired-end reads and supports either single or multiple genomes in one analysis. BacSeq can automatically complete assembling, annotating, identifying target genes, and analyzing comparative genomes. To start analysis using BacSeq, raw paired-end reads of the sample(s) are required. To run this tool, Conda (https://docs.anaconda.com/; accessed on 5 May 2023), an open-source package management system, is required to install BacSeq version 1.0 and all prerequisite software. BacSeq only supports Linux systems and requires a minimum space capacity of 1 Gb for installation.

### 2.3. Pipeline Customization

BacSeq offers two modes i.e., quick and advanced modes for novice and expert users, respectively. In quick mode, all results can be easily analyzed with a single click without further configuration. Default parameters are set in all tools in this mode for convenience. However, manual configuration can be used in the advanced mode. All bioinformatics tools can be configured parameters using a graphical user interface (GUI). In addition, expert users can optionally use any bioinformatics tools integrated into BacSeq via a command-line interface.

## 3. Results and Discussion

### 3.1. Graphical User Interface (GUI)

**Figure 2:**
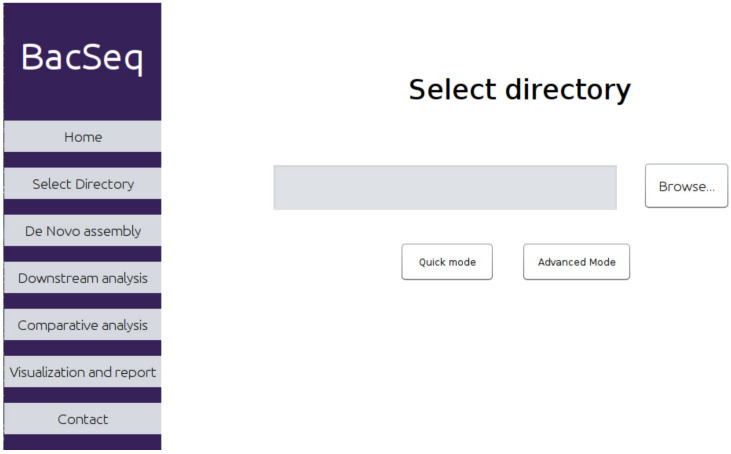
Graphical user interface (GUI) of BacSeq program.

The BacSeq pipeline was deployed as a JAVA-based application, enabling users to interact directly with the graphical user interface (GUI) for performing bioinformatics analysis, as shown in Figure 2. To start using BacSeq, users can simply select the directory containing the genome data and execute the program to complete the analysis in a single step. Users only need to use the command-line interface once for program installation. The pipeline supports both single files and multiple files in one analysis by just providing the absolute path of the directory of the file. However, all files must be prepared and renamed to an allowed pattern for the program to recognize the files and import them into the pipeline.

### 3.2. Use Case: Draft Genome Sequences of Acinetobacter baumannii Isolates

We used BacSeq to analyze the short-read sequencing data of 13 carbapenem-resistant *Acinetobacter baumannii* (CRAB) isolates, including PA020, PA025 (JAIGYO000000000), ST001 (JAIGSU000000000), ST009 (JAIGST000000000), ST010 (JAIGSS000000000), ST024 (JAIGSR000000000), ST028, ST032 (JAIGSQ000000000), ST034 (JAIGSP000000000), ST035 (JAIGSO000000000), ST036, YL005 (JAIGQD000000000), and YL006 (JAIGQC000000000). The qualified genomes of 10 isolates were deposited into the NCBI GenBank, except for the PA020, ST028, and ST036 isolates. Although the unqualified genomes existed in these 3 isolates, they were still included here as examples. The isolates were approved by the Human Research Ethics Committee (HREC) from Prince of Songkla University, Thailand (reference number: 64–284-14–1, date of approval: 9 June 2021). *A. baumannii* is a Gram-negative, rod-shaped, and aerobic bacterium that commonly causes hospital-acquired infection, particularly in intensive care units (ICUs) and among critically ill patients [33]. It has gained notoriety for its remarkable ability to acquire resistance to multiple antibiotics, especially carbapenem, through several mechanisms [33]. Moreover, their genetic materials, such as antibiotic resistance genes (ARGs) and virulence-associated genes (VAGs), could be transferred between the genus and other Gram-negative bacteria [34]. The Centers for Disease Control and Prevention (CD) have classified carbapenem-resistant *Acinetobacter* spp. as an urgent threat level [35]. Thus, the entire genome of this pathogen is necessary to be sequenced, which may provide more understanding of the genetic features related to its molecular evolution.

#### 3.2.1. Quality Control

**Figure 3:**
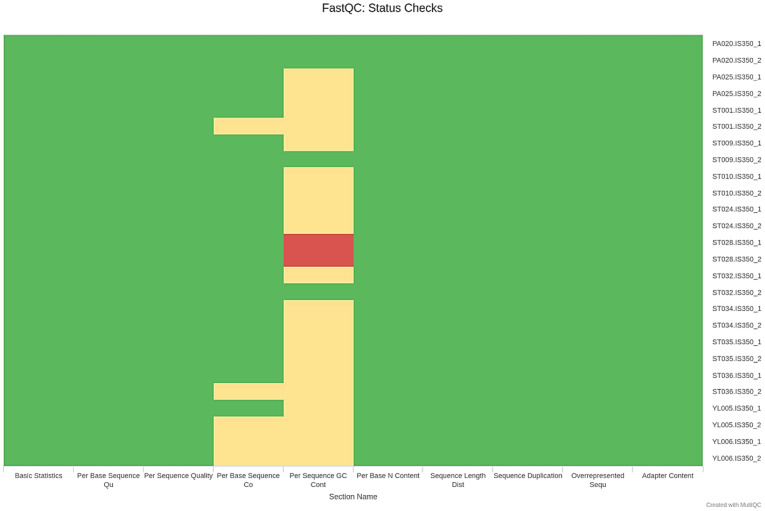
A report of status check by FastQC. Green, yellow, and red boxes represent pass, warn, and fail qualities, respectively.

According to the analysis workflow (Figure 1), quality control was initially performed to verify the raw reads. The reports exhibited total sequences, sequences flagged as poor quality, sequence length, %GC, total deduplicated percentage, average sequence length, basic statistics, Per base sequence quality, Per sequence quality scores, Per base sequence content, Per sequence GC content, Per base N content, sequence length distribution, sequence duplication levels, overrepresented sequences, and adapter content (Table S1). The results were reported as quantity or quality (pass, warn, and fail). As shown in Table S1 and Figure 3, the quality of most isolates was acceptable, while Per sequence GC content of the ST028 genome failed. This failure occurs when the cumulative deviations from the normal distribution of GC content in the reads exceed 30% [36].

#### 3.2.2. Genome Assembly, Assembly Quality Assessments, and Genome Annotation

**Figure 4:**
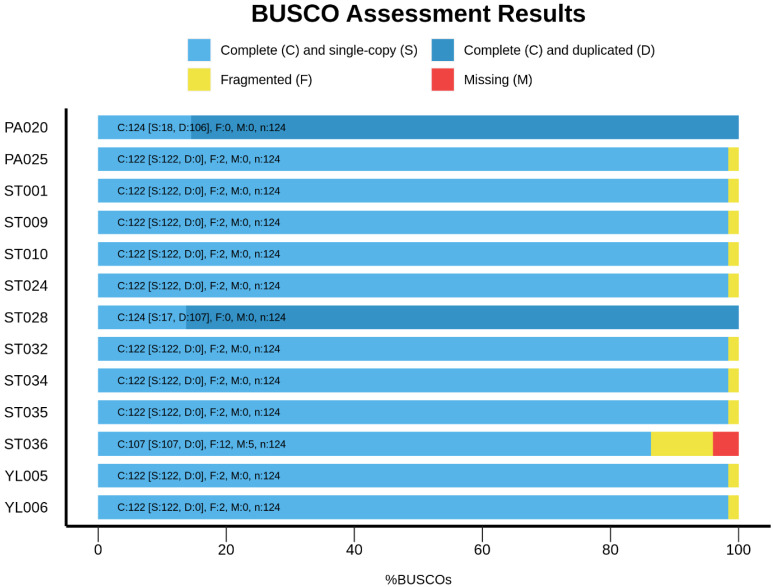
A report of genome completeness by BUSCO.

**Figure 5:**
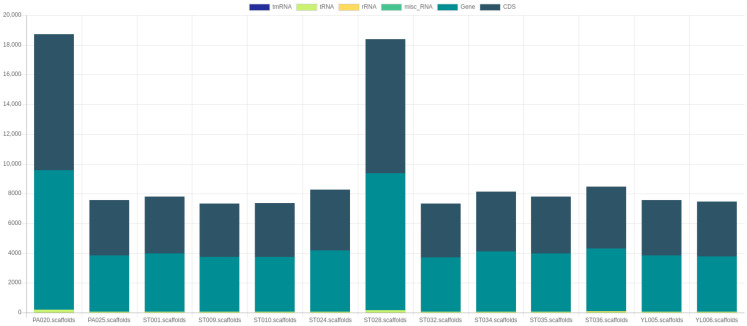
A report of genome annotation by Prokka.

The assembled sequences were subjected to quality assessments using QUAST, which reported the number of contigs, total sequence lengths, %GC, N50, N90, L50, and L90 (Table 1). We found that assembly of the ST036 genome provided a high number (*n* = 818) of total contigs, which generally indicates a more fragmented assembly. It means that the genome was not fully reconstructed into large, contiguous sequences but rather fragmented into numerous smaller pieces. This may occur due to various reasons, including repetitive or complex regions in the genome, sequencing errors, low coverage depth, or difficulties in resolving repetitive sequences [37]. However, a high number of contigs might be acceptable for some applications, such as comparative genomic analysis or identification of gene families; it is often desirable to have fewer contigs for a more complete and accurate representation of the genome. We also found that the PA020 and ST028 genomes consist of 9,243,789 bp and 9,539,281 bp, which is over the common length (approximately 3.7–4.4 bp) of the *A. baumannii* genome [38]. The reason may be the contamination of other bacterial genomes. However, these contaminant genomes were still included in further analyses, which could be used to compare it to other clean genomes. In addition, the completeness of assembled sequences was also assessed by BUSCO, which reported the percentages of complete, single-copy, duplicated, fragmented, and missing sequences (Figure 4). The result demonstrated that the duplicated sequences were observed in the PA020 and ST028 genomes, while the high percentages of fragmented and missing sequences were detected in the ST036 genome. For genome annotation, Prokka reports the amounts of tmRNA, tRNA, rRNA, miscRNA, gene, and coding sequence (CDS), as shown in Figure 5. Unusually high numbers of tRNA, genes, and CDS were observed in the PA020 and ST028 genomes due to their duplicated sequences. Additionally, Prokka also provided a GFF (General Feature Format) file that can be used as an input file in Roary for pan-genome analysis.

#### 3.2.3. Antibiotic Resistance, Also including Plasmids and Virulence Factors

**Figure 6:**
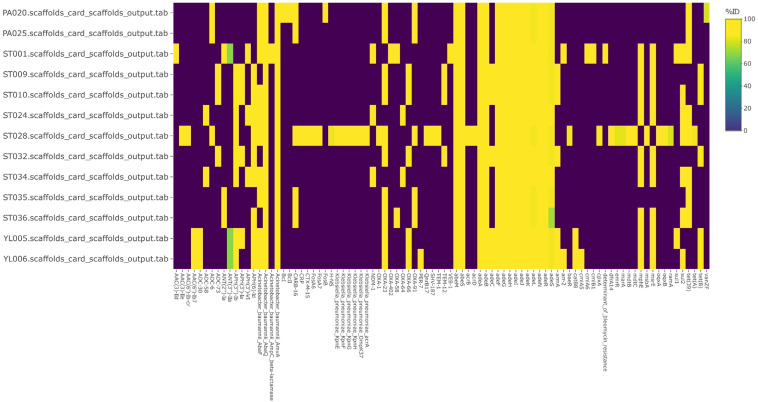
A report of antibiotic resistance genes (ARGs) against comprehensive antibiotic resistance database (CARD). ID, identity.

**Figure 7:**
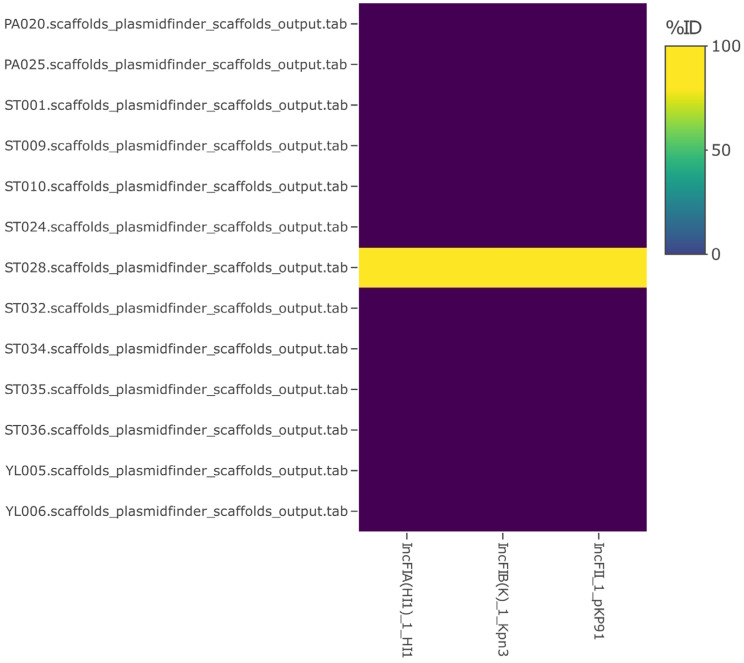
A report of plasmid types against PlasmidFinder database. ID, identity.

**Figure 8:**
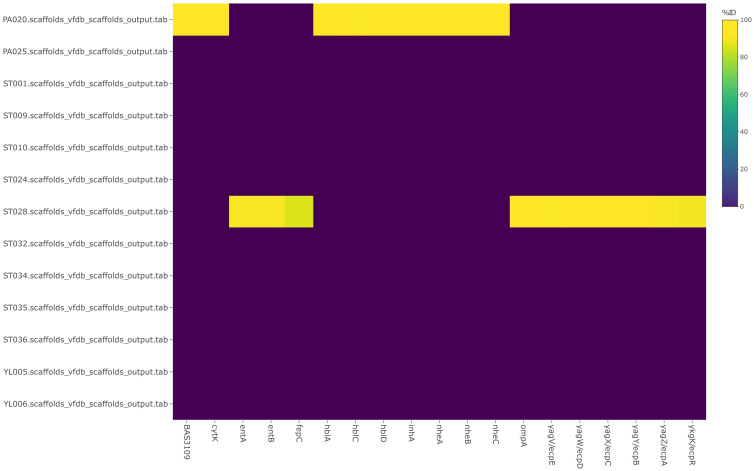
A report of virulence-associated genes (VAGs) against virulence factor database (VFDB). ID, identity.

For the identification of acquired ARGs, we provided an analysis against various databases in the ABRicate pipeline, including National Center for Biotechnology Information (NCBI), CARD, ResFinder, ARG-ANNOT, and MEGARes. Plasmid makers, VAGs, and sequence type (ST) could be also investigated, and their results were reported together with antibiotic resistance on the Hypertext Markup Language (HTML) page. In our case study, we reported the results of ARGs, plasmids, and VAGs, as illustrated in Figure 6, Figure 7 and Figure 8. We found that all clinical isolates of CRAB carried the genes that encoded for antibiotic efflux pumps conferring resistance to fluoroquinolone (*abaQ* and *abeM*), macrolide (*amvA* and *abeS*), and tetracycline (*adeA*, *adeB*, *adeL*, *adeR*, and *adeS*) (Figure 6). They also carried the genes that encoded for resistance-nodulation-cell division (RND) antibiotic efflux pump conferring multidrug resistance to tetracycline and fluoroquinolone (*adeF*, *adeG*, and *adeH*) and to rifamycin, diaminopyrimidine, penem, carbapenem, phenicol, tetracycline, macrolide, lincosamide, fluoroquinolone, and cephalosporin (*adeI*, *adeJ*, *adeK*, and *adeN*). In addition, antibiotic target alteration conferring aminoglycoside resistance (*armA*) and antibiotic inactivation (e.g., *bla*_OXA-23_, *bla*_NDM-1_, *bla*_CARB-16_, *aph(3’’)-Ib*, *aph(6’)-Id*, *mphE*, *msrE*, *fosA6*, and so on) were also detected in a high number of these CRAB isolates. Plasmid identification revealed that three plasmids, including IncFIA(HI1)_1_HI1, IncFIB(K)_1_Kpn3, and IncFII_1_pKP91, were only observed in the ST028 isolate (Figure 7). These plasmids, which have been classified as multidrug-resistant (MDR) plasmids, are commonly found in the Enterobacterales family, particularly *Salmonella* spp. and *Klebsiella* spp. [39,40,41,42], implying that the ST028 isolate may be contaminated with *Salmonella* spp. and/or *Klebsiella* spp. In virulence factor detection, we found that thiol-activated cytolysin gene (*BAS3109*), cytotoxin K (*cytk*), immune inhibitor A (*inhA*), hemolytic enterotoxin HBL complex genes (*hblA*, *hblC*, and *hblD*), and non-hemolytic enterotoxin genes (*nheA*, *nheB*, and *nheC*) were only harbored by the PA020 isolate (Figure 8). Enterotoxin genes (*entA*, *entB*, and *fepC*), outer membrane protein A gene (*ompA*), and adhesive virulence genes (*yagV/ecpE*, *yagW/ecpD*, *yagX/ecpC*, *yagY/ecpB*, *yagZ/ecpA*, and *ykgK/ecpR*) were only harbored by the ST028 isolate.

#### 3.2.4. Comparative Analysis

**Figure 9:**
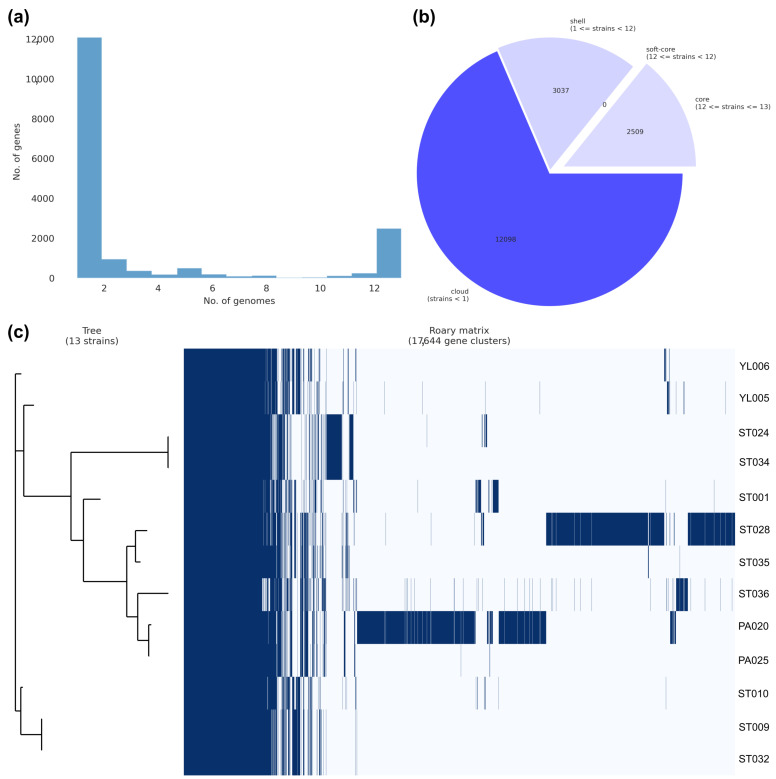
A report of pan-genome analysis by Roary. The frequency of genes versus the number of genomes (**a**), the breakdown of genes and the number of gene-presented isolates (**b**), and the phylogenetic tree against a matrix of present (blue) and absent (light blue) genes among core and accessory genomes (**c**) were exhibited.

In comparative genomic analysis, we provided Roary for analyzing pan-genome profiles among the studied genomes, which provide valuable insights into the genetic complexity and adaptability of species, helping us better understand their biology and evolution. For our case study, we found that 2509 (14.22%) core genes and 15,135 (85.78%) accessory genes were observed from 17,644 pan genes (Figure 9). Contaminant sequences in the PA020 and ST028 genomes resulted in the presence of high-level accessory genes existing in the pan-genome profile. The uncommon presence of these accessory genes could also be observed from the results of genome annotation and downstream analysis. In general, a large proportion of accessory genes in a pan-genome analysis can be indicative of an open pan genome, suggesting substantial genetic diversity within the studied population or species [43]. This observation further suggests the occurrence of horizontal gene transfer (HGT) events, facilitating the acquisition of novel genes from different isolates, species, or organisms [44,45]. Such pan-genome analysis provides valuable insights into the evolutionary history of the species, including ongoing speciation processes and the existence of subpopulations harboring distinct gene sets [44,45].

#### 3.2.5. Other Analysis

In BacSeq, we also provide bioinformatics tools for identifying CAZyme and CRISPR-Cas systems, and the results are reported in the Other Analysis button. The CAZyme reported annotated genes that encoded for the families of carbohydrate-active enzymes. Carbohydrate-active enzymes are enzymes involved in the breakdown, biosynthesis, or modification of carbohydrates, and they play a crucial role in various biological processes, including digestion, microbial metabolism, and the degradation of complex carbohydrates [46]. This tool is beneficial for analyzing WGS data of potential bacteria, especially probiotic strains. It can provide insights into their ability to metabolize and interact with different carbohydrates. This information is valuable for understanding the potential health benefits of probiotics, as carbohydrates are a significant component of the diet and can influence various aspects of human health, including the gut microbiota composition and metabolic activities [47,48]. Meanwhile, CRISPRCasFinder reported the location of CRISPR-Cas systems (bacterial adaptive immune system), which included the sequences of direct repeats and spacers as well as the types of Cas gene groups. The presence of this system could imply the adaptive evolution of the studied genomes to foreign genetic elements, especially bacteriophage genomes [49]. This tool is good for bacterial evolution study as well, as it could be the model of genetic engineering [50,51]. Furthermore, the identification of bacteriocin-encoding genes and restriction–modification (R-M) sites will be implemented in the future versions of BacSeq.

### 3.3. Hybrid Library Assembly for Complete Genome Analysis

Long-read WGS is commonly beneficial for studying the complete genome of particular bacteria because it can be used to distinguish bacterial chromosome(s) and plasmids. The analytical steps in BacSeq were almost similar, except for genome assembly. Here, we used Unicycler for assembling the bacterial genome using both SRS and LRS data with a hybrid assembly method. The complete genome probably includes the chromosome and plasmids, which can be separated into distinct contigs or fragments. The separation of chromosome and plasmid sequences within a complete genome assembly enhances the understanding of the bacterial genomic structures, facilitates comparative genomics studies, enables functional analysis of plasmid-borne elements, and supports various applications in genetic engineering and biotechnology.

### 3.4. Limitation of BacSeq

In this study, we provide BacSeq as an automated pipeline for analyzing WGS data of bacterial genomes. However, the main limitation of BacSeq is that when the user runs the program and contaminant sequences occur in the analysis, the program cannot exclude contaminating sequences from the studied genome. We suggest using Kraken [52] or other tools to identify the contaminated sequences and remove them from the analysis, as demonstrated in our previous study [38]. Nevertheless, we recommend that the researcher avoid the contamination in the bacterial culture and pick a single isolated pure colony for genomic DNA extraction before performing WGS and bioinformatics analysis. This limitation will be addressed in future versions of BacSeq.

## 4. Conclusions

BacSeq is an open-source comprehensive pipeline integrating various bioinformatics tools for analyzing WGS data of bacterial genomes that research communities can easily install and implement on laptops and high-performance computers. BacSeq provided an automated bioinformatics workflow starting from genome assembly, annotation, and antimicrobial resistance gene identification to comparative genome analysis. Furthermore, BacSeq can generate comprehensive reports and plots in a web form which could help users simply explore and extract interesting information from the analysis.

## Figures and Tables

**Table 1:** A report of quality assessments by QUAST.

Isolate Code	Number of Contigs	Total Length	%GC	N50	N90	L50	L90
PA020	92	9,243,789	36.80	569,197	59,780	6	29
PA025	66	3,906,279	38.89	152,139	41,611	6	24
ST001	68	4,111,741	39.02	128,875	39,322	8	28
ST009	69	3,844,585	39.01	113,438	40,316	11	32
ST010	68	3,872,017	38.93	123,627	42,752	10	30
ST024	62	4,294,911	38.82	250,119	64,045	6	18
ST028	187	9,539,281	49.62	187,251	35,730	17	58
ST032	67	3,844,381	39.01	122,061	42,602	11	31
ST034	58	4,225,388	38.88	250,219	71,864	6	16
ST035	70	4,035,126	38.99	176,611	43,801	7	25
ST036	818	4,329,065	38.79	65,441	1278	15	255
YL005	109	3,910,735	38.91	76,044	195,99	18	55
YL006	53	3,894,856	38.99	190,977	61,096	6	19

## Data Availability

The BacSeq pipeline with its detailed user manual is publicly available at https://github.com/mecobpsu/bacseq (accessed on 5 May 2023).

## Supplementary Materials

The following supporting information can be downloaded at: https://www.mdpi.com/article/10.3390/microorganisms11071769/s1, Table S1: a report of FastQC results.
